# Targeting the Antibody Checkpoints to Enhance Cancer Immunotherapy–Focus on FcγRIIB

**DOI:** 10.3389/fimmu.2019.00481

**Published:** 2019-03-12

**Authors:** Ingrid Teige, Linda Mårtensson, Björn L. Frendéus

**Affiliations:** BioInvent, Lund, Sweden

**Keywords:** therapeutic antibody, antibody checkpoint, fc gamma receptor, cancer immunotherapy, drug resistance, tumor microenvironment

## Abstract

Immunotherapy with therapeutic antibodies has increased survival for patients with hematologic and solid cancers. Still, a significant fraction of patients fails to respond to therapy or acquire resistance. Understanding and overcoming mechanisms of resistance to antibody drugs, and in particular those common to antibody drugs as a class, is therefore highly warranted and holds promise to improve response rates, duration of response and potentially overall survival. Activating and inhibitory Fc gamma receptors (FcγR) are known to coordinately regulate therapeutic activity of tumor direct-targeting antibodies. Similar, but also divergent, roles for FcγRs in controlling efficacy of immune modulatory antibodies e.g., checkpoint inhibitors have been indicated from mouse studies, and were recently implicated in contributing to efficacy in the human clinical setting. Here we discuss evidence and mechanisms by which Fc gamma receptors–the “antibody checkpoints”–regulate antibody-induced antitumor immunity. We further discuss how targeted blockade of the sole known inhibitory antibody checkpoint FcγRIIB may help overcome resistance and boost activity of clinically validated and emerging antibodies in cancer immunotherapy.

## Introduction

Monoclonal antibody-based therapies have revolutionized cancer treatment improving survival for patients with hematologic and solid cancers. The clinically most successful antibodies exert antitumor activity either by targeting tumor cells directly (direct-targeting antibodies) (1–4), or by targeting and activating immune cells that seek up and kill cancer cells in the tumor microenvironment (immune checkpoint antibodies) (5–13).

While both types of mAb are highly potent with cancer curative potential a significant fraction of patients fail to respond or develop resistance to treatment (14–17). An improved understanding of mechanisms underlying resistance, and in particular those common to antibody drugs as a class–including direct-targeting and immune checkpoint antibodies–is needed for rational development of drugs that could help boost efficacy, and prevent or overcome antibody drug resistance. Given the broad use of antibodies in cancer treatment, such drugs would have the potential to fundamentally improve cancer survival.

### FcγR Regulation of Antibody-Induced Immunity–“The Antibody Checkpoints”

The Fc receptors (FcR) are the only receptors of the immune system known to regulate the activity of antibodies as a class (18). FcRs orchestrate antibody-induced effector cell responses and immunity through low affinity, high avidity interactions with aggregated antibody Fc-domains of antibody-coated cells or immune complexes, generated following antibody Fv-binding to target receptors. Because Fc domains are conserved between antibodies of a given subclass e.g., IgA, IgE, IgM, or IgG_1_, IgG_2_, IgG_3_ or IgG_4_, FcRs regulate antibody-induced immune responses irrespective of antigen specificity. For this same reason FcRs regulate immune responses induced both by endogenously generated antibodies (e.g., antibodies mounted in response to infection or underlying inflammatory or autoimmune disease) and recombinantly produced therapeutic monoclonal antibodies (18, 19). Of particular relevance for cancer immunotherapy the Fc gamma receptors (FcγR) are known to regulate the activity of Immunoglobulin G type of antibodies (20), the group to which all antibodies approved for cancer therapy belong.

**Figure 1:**
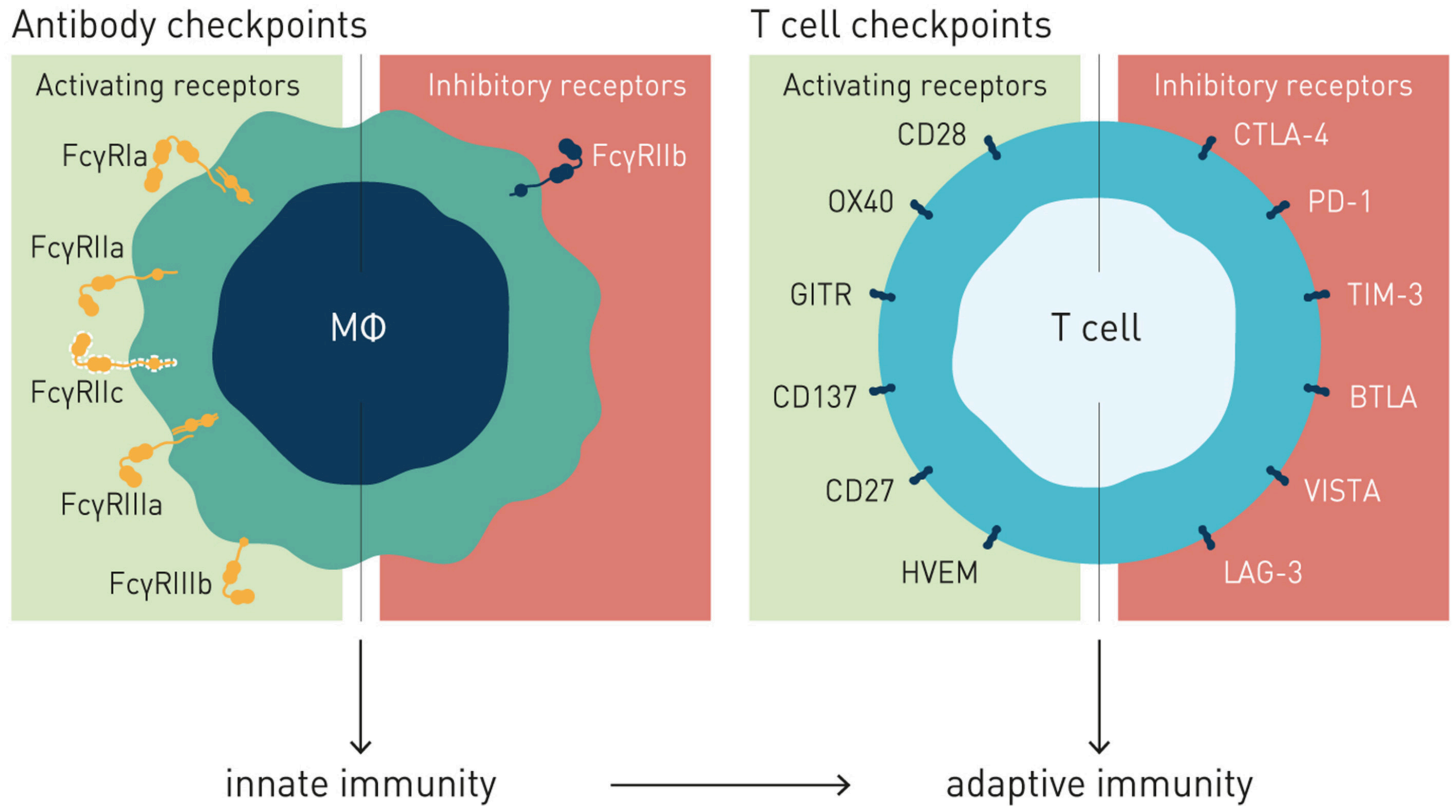
Antibody and T cell checkpoints. Both T cell and antibody checkpoints comprise activating (co-stimulatory) and inhibitory receptors. However, antibody checkpoints are co- expressed only on innate immune cells e.g., macrophages and dendritic cells, and comprise only a single inhibitory member (FcγRIIB).

The family of FcγRs share several characteristics with the T cell immune checkpoints in how they regulate effector cell activation and immune responses (Figure 1). Recent work by ourselves and others, reviewed in detail below, demonstrate a critical role for this receptor family as concerted regulators of antibody-induced innate and adaptive immunity. Consequently, the FcγRs are therapeutically important immune checkpoints, and since they control immune activity of IgG antibodies as a class, we propose to refer to them as “antibody checkpoints.” We will herein use antibody checkpoint and FcγR interchangeably.

### Antibody and T Cell Checkpoints–Similarities and Differences

**Figure 2:**
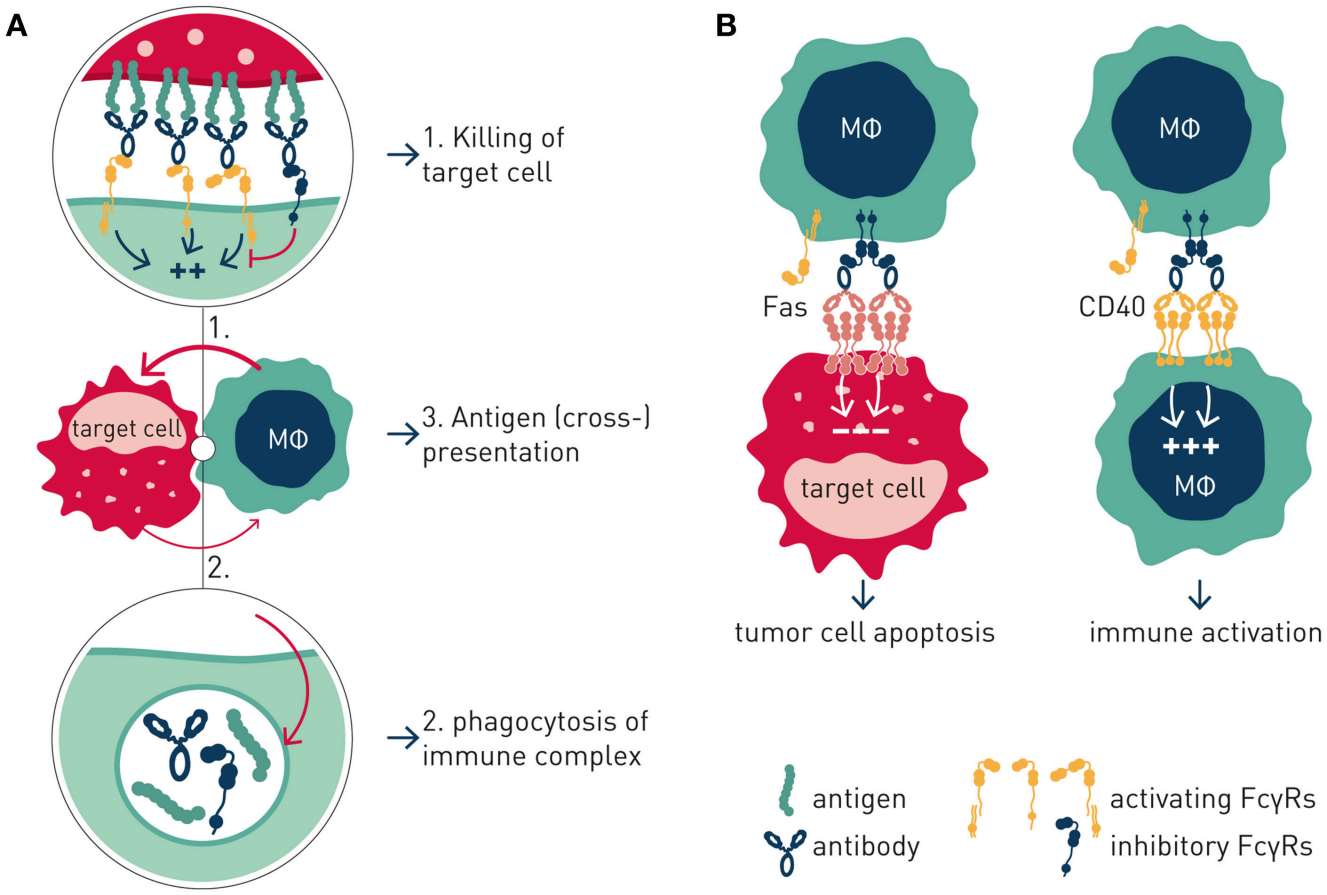
Antibody checkpoint intrinsic and extrinsic signaling. **(A)** Intrinsic signaling. Antibody checkpoints relay aggregated antibody Fc-induced signals into effector cells (MΦ) in a concerted manner through ITAM containing activating (aFcγR) and ITIM-containing inhibitory (iFcγR) Fc gamma receptors. FcγR-expressing cell responses include phagocytosis, immune complex endocytosis, and antigen presentation. **(B)** Extrinsic signaling. Antibody checkpoints promote clustering and signaling induced by antibody targeted receptors in an antibody Fv and Fc co-dependent manner. Cellular responses are determined by the antibody-targeted receptor's function e.g., macrophage co-stimulation or tumor cell apoptosis.

Like the T cell checkpoints the Fc gamma receptors (FcγR) fall into either of two functionally distinct groups, which coordinately regulate immune effector cell activation and ensuing immune responses (Figure 1). Activating FcγR, like co-stimulatory T cell checkpoints, promote effector cell activation, and immunity. In contrast, inhibitory FcγR, like the T cell co- inhibitory checkpoints, block cellular activation and down-modulate immune responses. Adding to complexity, antibody checkpoints may–similar to the T cell checkpoints–promote checkpoint receptor extrinsic signaling by facilitating cross-linking and signaling of ligand receptors (21, 22). In case of the antibody checkpoints, this would equate to FcγR-mediated cross-linking of antibody Fv-targeted receptors (Figure 2). Depending on ligand receptor function, such signaling may be activating or inhibitory, as has been described for agonistic CD40 and agonistic Fas antibodies, respectively (23–27). FcγR extrinsic signaling may, or may not, contribute to therapeutic efficacy.

The activating and the inhibitory FcγR receptors transmit their signals into FcγR-bearing immune cell via immunoreceptor tyrosine-based activation motifs (ITAM), and immunoreceptor tyrosine-based inhibitory motifs (ITIM), respectively. Specifically, how target cell-bound antibodies modulate immune cell activation is determined by their relative engagement of activating and inhibitory Fcγ receptors. This in turn is determined by the size of the FcγR-engaging immune complex, i.e., the number of antibodies coated onto a target cell (determined by cellular expression levels of antibody targeted receptor), availability of activating and inhibitory Fcγ receptors, and antibody isotype. Different antibody isotypes bind with different affinity to activating and inhibitory Fcγ receptors, resulting in different activating: inhibitory (A:I) ratios, and differential ability to mediate e.g., activating FcγR-dependent target cell deletion (28) or inhibitory FcγR-dependent agonism (23, 24).

As in the T cell checkpoint family, there are several activating antibody checkpoints that individually, and collectively, positively regulate antibody-induced cell activation. In humans, the activating FcγR's are: FcγRI (CD64), FcγRIIa (CD32a), FcγRIIc (CD32c), and FcγRIIIa (CD16a) (29, 30). The GPI-linked FcγRIIIb lacks an intracellular signaling domain and ITAM motifs, but is nevertheless often considered an activating FcγR, since it has been shown to promote neutrophil activation and effector cell mediated target cell killing in response to challenge with antibody-coated target cells (31, 32). The activating mouse FcγRs are: FcγRI, FcγRIII, and FcγRIV (28, 30, 33).

Most Fc gamma receptors bind monomeric IgG with low to intermediate (μM) affinity [as reviewed in detail elsewhere (28, 29, 33)]. Immune complex formation allows for high-avidity binding of multimerized IgG Fc's to the low-affinity FcγRs, which are cross-linked, leading to FcγR-expressing cell activation. In contrast, free circulating IgG has too low affinity to promote stable Fc:FcγR binding, and cannot promote FcγR-cross-linking, or cell activation. How high affinity FcγRs e.g., FcγRI and mouse FcγRIV, which may bind monomeric uncomplexed IgG, sense and trigger activation in response to immune complexes and antibody-coated cells remains a subject of debate. It is however clear that the high affinity FcγRs may critically contribute to therapeutic antibody efficacy and pathology (33, 34).

Multiple isoforms and allelic variants of the individual FcγRs are known, and the affinities of the clinically most significant variants for different human IgG subclasses have been described (29). Of particular significance for cancer immunotherapy, two isoforms of the low and intermediary affinity antibody checkpoints FcγRIIa (H131R) and FcγRIIIa (V158F), which bind IgG and antibody-coated target cells with higher affinity and avidity, have been associated with improved survival of diverse cancer patients in response to antibody-based cancer immunotherapy (35–39). These, and additional polymorphisms of low and intermediary affinity activating and inhibitory FcγRs, which alter affinity for IgG, or modulate FcγR expression levels, are further associated with susceptibility to antibody-mediated chronic inflammatory and autoimmune disease (40). Of further functional consequence, there is extensive gene copy number variation in high and low affinity loci that affect expression levels of individual FcγRs (41–43).

The antibody checkpoints differ from the T cell checkpoints in notable and critical aspects, which have important consequences for the type of immune response induced, and for design of drugs aimed at harnessing and enhancing FcγR-mediated immunity (Figure 1).

Firstly, in contrast to the T cell checkpoints the Fc gamma receptors are not generally expressed on T cells, but principally on cells of the innate immune system, and in a restricted manner on B cells (FcγRIIb) and NK cells (FcγRIIIa and FcγRIIc, the latter in ~20% of caucasians) (18, 30, 41). In particular cells specialized in MHC class II-restricted antigen presentation, e.g., macrophages and dendritic cells, express both activating and inhibitory FcγRs, enabling fine-tuned regulation of antibody-induced immune responses (28, 44). Consequently, the antibody checkpoints hold the key to unleash antibody-induced immunity first and fore-most through improving innate immune effector mechanisms, e.g., macrophage dependent phagocytosis (ADCP), and dendritic cell mediated antigen presentation, and cross-presentation (45–51). Triggering and enhancing innate immune activation and robust antigen presentation is known to critically contribute to and underlie robust adaptive T cell-mediated antitumor responses, including those induced by antibodies targeting T cell checkpoints (18, 52–54). Modulation of antibody checkpoints therefore has the potential to improve also adaptive antitumor responses, possibly decreasing the threshold of tumor mutational burden for cancers to respond to antibody-mediated cancer immunotherapy (55). Finally, and in stark contrast to the multiple inhibitory T cell checkpoints described, only a single inhibitory antibody checkpoint–Fc gamma receptor IIB–is known (Figure 1).

## Antibody Checkpoints Determine Anti-Cancer Antibody Efficacy

### Cancer Cell Direct-Targeting Antibodies

The CD20-specific antibody rituximab was the first antibody to be approved by the FDA for cancer therapy and is arguably the clinically best validated antibody used in cancer immunotherapy. As such rituximab provides a prime example of a tumor cell direct-targeting antibody that has been exhaustively studied from a mechanism-of-action perspective. While multiple mechanisms, including induction of apoptosis and triggering of complement mediated cell lysis, have been proposed to contribute to and underlie rituximab therapeutic activity (56, 57), the strongest preclinical, and clinical evidence point to Fc gamma receptor dependent mechanisms (58–61).

Independent retrospective studies have established a correlation between one or more activating Fc gamma receptors and clinical efficacy in different types of lymphoma. Patients homozygous for high affinity allelic variants of the activating antibody checkpoints FcγRIIIa or FcγRIIa showed improved responses and survival in response to rituximab therapy compared to patients carrying one or more lower affinity alleles (35, 36). Similar links between response and FcγR-dependent mechanisms have been observed for additional cancer cell direct-targeting antibodies e.g., herceptin (anti-Her2) and cetuximab (anti-EGFR) in breast cancer (38) and colorectal patients, respectively (37, 39). These observations have spurred biotech and pharmaceutical companies to engineer antibodies with improved binding to activating antibody checkpoints. Obinutuzumab, a glycoengineered antibody with improved affinity for FcγRIIIa, was approved for clinical use based on increased overall survival in a head-to-head comparison with rituximab in CLL patients (15). Taken together, these observations demonstrate that antibody checkpoints can determine clinical efficacy of cancer cell direct-targeting antibodies.

Consistent with the well-conserved function of activating and inhibitory antibody checkpoints between mouse and man, similar dependencies between activating FcγRs and cancer cell direct-targeting antibodies have been made in mouse cancer experimental models. Further in keeping with common, ITAM-signaling dependent, functions of the several activating antibody checkpoints, genetic ablation of individual activating FcγRs typically has shown limited effects on *in vivo* therapeutic efficacy compared to ablation of all activatory FcγRs (28, 33, 62).

In stark contrast, genetic deletion of the sole inhibitory antibody checkpoint FcγRIIB fundamentally enhances *in vivo* therapeutic activity of cancer cell direct-targeting antibodies, including those specific for CD20, Her2, and EGFR i.e., clinically validated targets in therapy of hematologic malignancy as well as solid cancer (63). These observations indicate the significant therapeutic potential of targeting the inhibitory antibody checkpoint, and indicate that redundancy needs to be accounted for when seeking to enhance antibody efficacy by modulating activating antibody checkpoints, much as has been observed in targeting of the multiple different T cell checkpoints (6, 14, 64).

**Figure 3:**
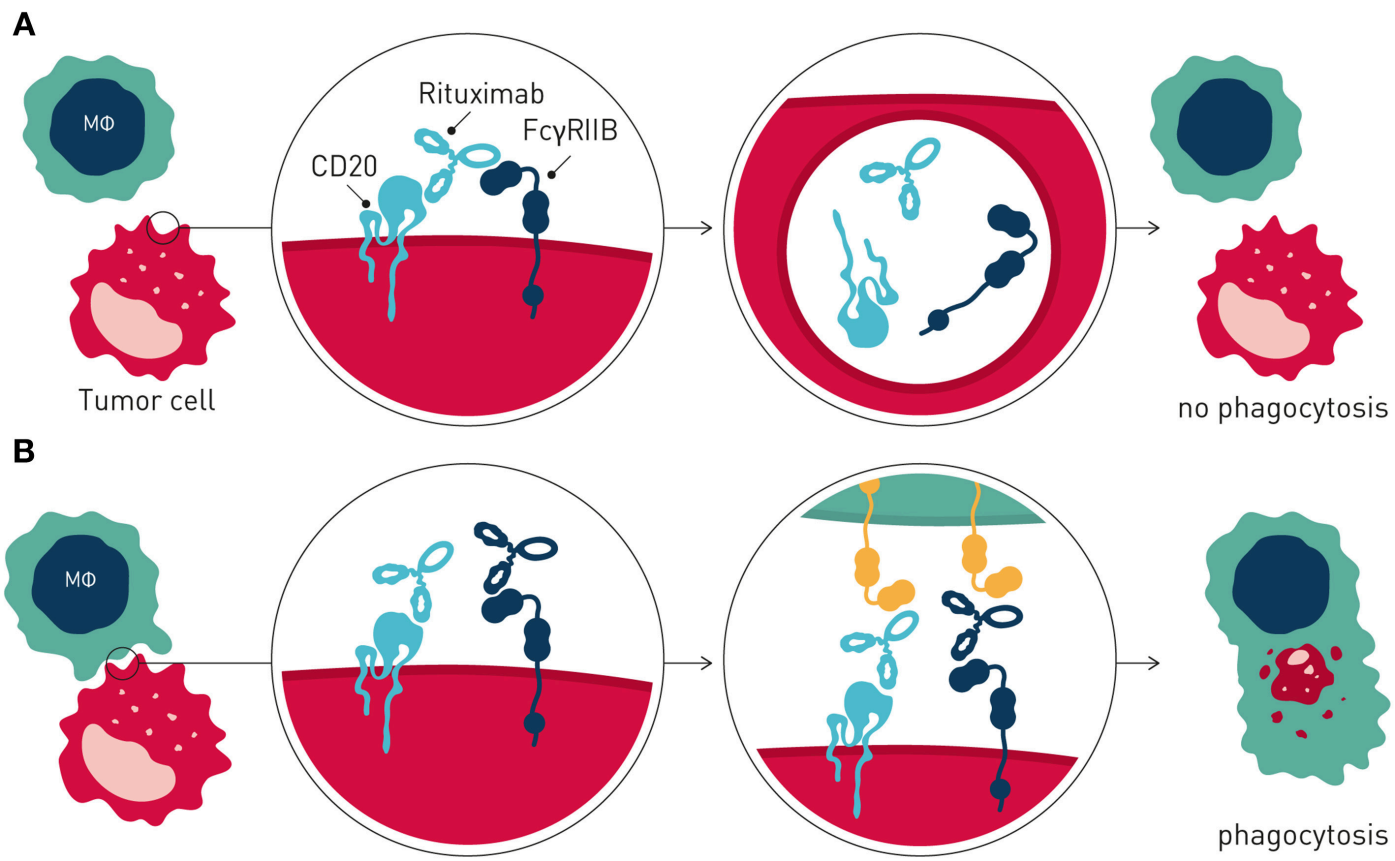
Tumor cell expressed FcγRIIB promotes antibody drug resistance. **(A)** Resistance is mediated by FcγRIIB-mediated removal of antibody molecules from the tumor cell surface through a process of internalization. **(B)** Blocking antibodies to FcγRIIB prevent internalization, leaving greater numbers of therapeutic antibody on the tumor cell surface, promoting immune effector cell-mediated antitumor activity.

Interestingly, and in further support of FcγRIIB being a tractable target in cancer immunotherapy, recent data has demonstrated that this inhibitory antibody checkpoint limits therapeutic antibody efficacy and promotes antibody drug resistance by additional mechanisms distinct from inhibitory signaling in immune effector cells, when expressed on tumor B cells (65) (Figure 3). Beers et al. found that FcγRIIB expressed on tumor B cells promoted internalization of rituximab antibody molecules from the tumor B cell surface, increasing antibody consumption and leaving fewer rituximab molecules to engage critical FcγR-dependent effector cell-mediated antitumor activity e.g., ADCP (66). FcγRIIB expression correlated with rituximab internalization across several different lymphoma subtypes studied. Highest and most homogenous expression of FcγRIIB is observed in Chronic Lymphocytic Leukemia (CLL), Mantle cell lymphoma (MCL), and Marginal Zone Lymphoma, although a fraction of Follicular lymphoma (FL) and Diffuse Large B cell Lymphoma show exceptionally high FcγRIIB expression (67, 68). Further consistent with tumor B cell expressed FcγRIIB limiting antibody therapeutic efficacy and promoting antibody resistance, retrospective clinical studies of MCL and FL patients treated with rituximab-containing therapy showed decreased survival of patients with higher FcγRIIB expression on tumor cells (67, 69). Tumor cell expressed FcγRIIB appears to be a general mechanism limiting antibody therapeutic efficacy and promoting antibody drug resistance in the tumor microenvironment. Using a humanized model of treatment refractory B cell leukemia, and the CD52-specific antibody alemtuzumab, Pallasch et al. found that FcγRIIB is highly overexpressed on leukemic tumor cells in such antibody drug-resistant tumor microenvironments, and that shRNA-mediated knock-down of tumor cell FcγRIIB restored responsiveness to therapeutic antibody resulting in animal cure (70). Finally, high expression of FcγRIIB in B cell malignancy may indicate that immunocompetent antibodies to FcγRIIB could have single agent therapeutic activity in this setting (65, 71).

Collectively, these and other observations provided the rationale to develop antagonistic anti-FcγRIIB antibodies that block FcγRIIB-mediated antibody internalization for combination immunotherapy of B cell cancer with direct-targeting antibodies e.g., rituximab (65, 72) (Figure 3).

### Antibodies to Immune Checkpoint Inhibitory Receptors

Antibody targeting of immune inhibitory T cell checkpoints e.g., CTLA-4, PD-1 and PD-L1 has transformed solid cancer therapy shifting focus from cancer cell-direct targeting therapies to immune modulatory drugs, which induce long-term remission and apparent cures albeit in a small fraction of advanced stage cancer patients. Such immune checkpoint-directed therapy has increased overall survival for patients with various cancers, notably including multiple solid cancer types e.g., melanoma, lung, bladder, and head and neck cancer, and are approved by the Food and Drug Administration (14, 73, 74).

While originally thought to act solely via “blocking the brake” on effector T cells (74, 75), recent preclinical and clinical data indicate a critical role for FcγR's in regulating therapeutic efficacy of antibodies to inhibitory T cell checkpoints. Vargas et al. for the first time in human subjects, demonstrated a link between antibody checkpoints, and clinical response to T cell checkpoint targeted antibody therapy (76). Melanoma patients carrying a high affinity allele of the activating FcγRIIIa (V158) showed improved survival in response to treatment with the anti-CTLA-4 antibody ipilimumab compared to patients carrying a lower affinity FcγRIIIa (F158) allele. Interestingly, in the two retrospectively studied cohorts, a prerequisite for response to anti-CTLA-4 antibody therapy was that patients had inflamed tumors i.e., T cells had infiltrated tumors prior to commencing therapy. The observation that antibody checkpoints determine clinical efficacy of ipilimumab was not unexpected, since anti-CTLA-4 antibody therapy in the mouse critically depends on FcγR-mediated deletion of regulatory T cells (77–80), which express CTLA-4 at higher levels compared with effector T cells in the tumor microenvironment (76). Consistent with co-ordinate regulation of anti-CTLA-4 antibody therapeutic efficacy by the antibody checkpoints, in a FcγR-humanized mouse model antibody variants engineered for enhanced binding to activatory FcγR showed enhanced therapeutic activity (76). In contrast, antibody variants with diminished binding to activating FcγR failed to induce protective immunity against cancer.

So, how about the other clinically validated T cell checkpoints? Do antibody checkpoints regulate the activity also of antibodies targeting the PD-1/PD-L1 axis? Evidence from mouse models suggests that indeed they do. Interestingly, however, these data indicate differential FcγR-regulation for anti-PD-1 and anti-PD-L1 antibodies. Dahan et al. reported that anti-PD-L1 antibodies therapeutic efficacy was enhanced with antibody isotypes that preferentially engage activating over inhibitory antibody checkpoints (81). Conversely, anti-PD-1 antibody variants that did not engage FcγRs showed greatest therapeutic activity, and FcγR-engaging antibodies' activity decreased with increasing A:I ratios. Similarly, Pittet and coworkers found that *in vitro* and *in vivo* efficacy of clinically approved anti-PD-1 antibodies nivolumab and pembrolizumab, and a murine surrogate antibody variant with claimed similar engagement of mouse FcγR compared to these mAb, was compromised by FcγR-engagement (82). Deglycosylation of antibodies with EndoS rendering them incapable of engaging FcγRs, or antibody-mediated FcγR-blockade, significantly improved anti-PD-1 antibody therapeutic activity. This demonstrates that FcγRs negatively regulate anti-PD-1 antibody efficacy. Further studies are needed to dissect the relative importance of activating vs. inhibitory antibody checkpoints in regulating anti-PD-1/PD-L1 antibodies' therapeutic activity.

### Antibodies to Immune Checkpoint Co-stimulatory Receptors

The power of treating cancer by engaging patient's own immune defense mechanisms through immunotherapy with antibodies to the co-inhibitory T cell checkpoints, has prompted the question of whether targeting also co-stimulatory immune checkpoints e.g., 4-1BB, OX40, CD40, and GITR can translate into similarly efficacious and perhaps complementary pathways of anti-cancer immunity?

Preclinical and limited clinical data has indicated both single agent activity of antibodies to co-stimulatory immune checkpoints and complementary effects following combination with checkpoint blocking antibodies e.g., anti-PD-1 (83–89). As found for antibodies to the immune inhibitory checkpoints, and as discussed below, efficacy of immune agonist checkpoint antibodies is regulated by the FcγRs (77–79, 89), with some showing preferential engagement of activatory FcγR (i.e., high A:I ratio), and others of inhibitory FcγR (i.e., low A:I ratio), for optimal therapeutic activity (Table 1).

**Table 1:** Antibody checkpoints determine efficacy and mechanism-of-action of immune modulatory antibodies.

**Antibody MoA**		**Co-stimulatory checkpoints**					**Co-inhibitory checkpoints**		
		**GITR**	**OX40**	**4-1BB**	**CD40**	**IL-2R**	**CTLA-4**	**PD-1**	**PD-L1**
High A:I ratio	Effect	Treg depletion			CD40^+^ cell depletion	Treg depletion	Treg depletion	*FcγRs reduce efficacy	TAM depletion?
	FcγR-modulation	aFcγR↑ iFcγR↓	aFcγR↑	aFcγR↑ iFcγR↓	aFcγR↑ iFcγR↓	aFcγR↑ iFcγR↓	aFcγR↑ iFcγR↓		aFcγR↑ iFcγR↓
Low A:I ratio	Effect	Teff costimulation			APC costimul.				
	FcγR-modulation		aFcγR↓ iFcγR↑	aFcγR↓ iFcγR↑	aFcγR↓ iFcγR↑				
FcγR-indep.	Effect						Block Teff suppression		
mAbs	Isotype(s)	rIgG2b	mIgG1	mIgG2a, mIgG1	mIgG1, hIgG1/2/SE/ SELF/V9/V11	rIgG1, mIgG2a	haIgG, hIgG1	mIgG1/2a/ 1_D265A_, rIgG1, hIgG4	mIgG1/2a/ 1_D265A_
	Clone(s)	DTA-1	OX86	LOB12.0	1C10, 3/23, FGK45, CP-870,893	PC-61	9H10, 4F10, 9D9, ipilimumab	4H2, RPMI-14, nivolumab, pembro	14D8

*Table indicates antibody Mechanism-of-Action (**MoA**) as a function of antibody isotype preferential engagement of activating (**High A:I ratio**) or inhibitory (**Low A:I ratio**) antibody checkpoints. Mechanisms of immune modulatory antibodies to co-stimulatory immune checkpoints, co-inhibitory immune checkpoints or the IL-2R are indicated. **Effect** indicates main cell type and function identified as underlying therapeutic effects of High A:I, and Low A:I variant antibodies, respectively. **FcγR-modulation:** arrows indicate how activatory FcγR (aFcγR) and inhibitory FcγR (iFcγR) positively (↑) or negatively (↓) regulate indicated effect. Bottom two lines indicate antibody isotypes and clones used in referenced studies. **References**: **GITR** (79), **OX40** (90–95), **4-1BB** (89), **CD40** (23, 24, 96), IL-2R (97), **CTLA-4** (76–80), **PD-1** (81, 82), **PD-L1** (81)*.

**Figure 4:**
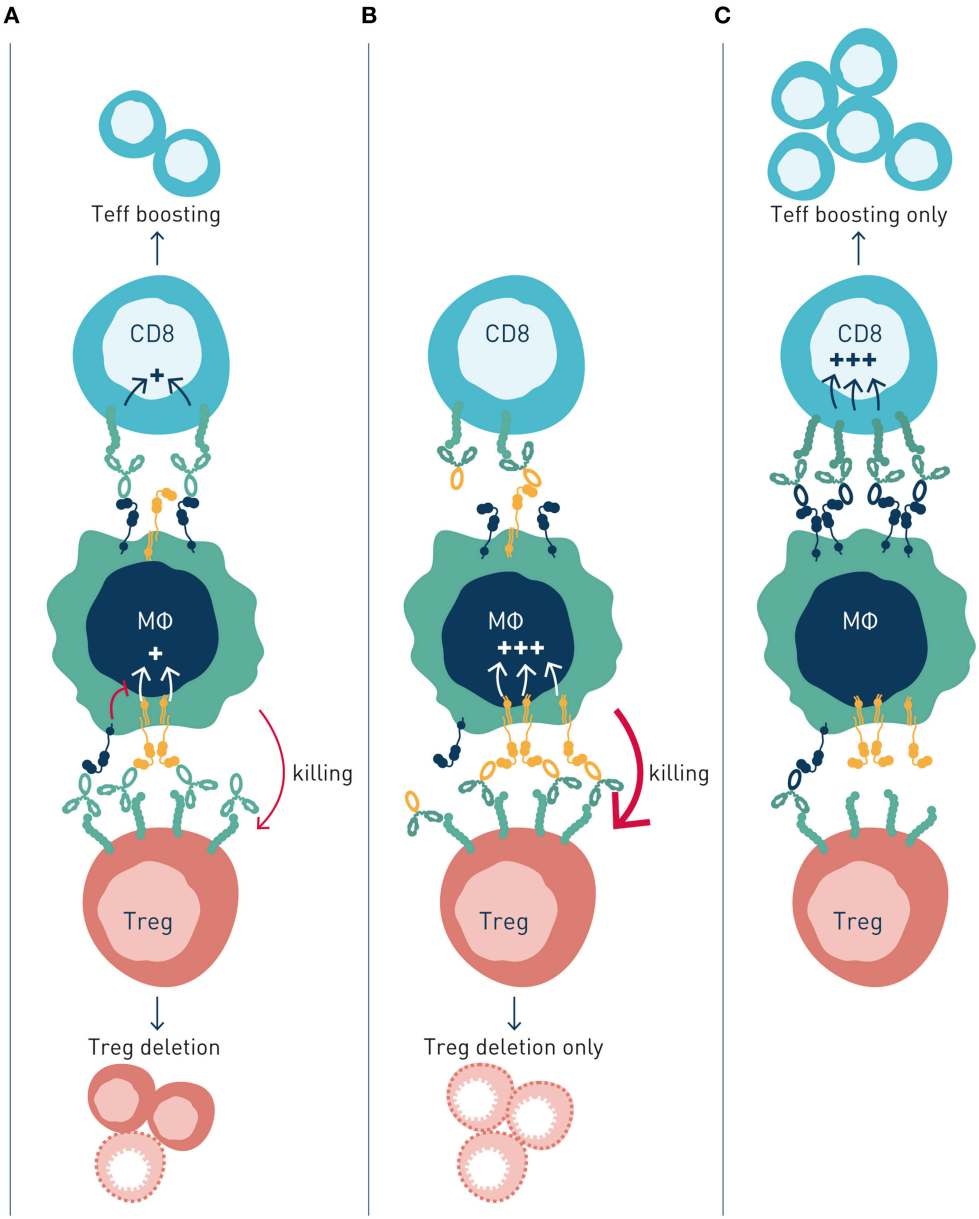
Activating and inhibitory antibody checkpoints determine efficacy and mechanism-of-action of immune agonist antibodies. This schematic figure models **(A)** Antibody engagement of activating and inhibitory FcγRs determine target cell depletion and agonism, respectively. The two mechanism compete when antibody variants (isotype) capable of binding both FcγR are used, resulting in reduced or no therapeutic activity. **(B)** Antibody variants with enhanced binding to activating FcγR (high A:I ratio) show improved depletion of Treg cells, which express higher numbers of receptors compared with effector cells, resulting in immune activation through elimination of suppressor cells. **(C)** Antibody variants with enhanced binding to inhibitory FcγR (low A:I ratio) show improved CD8^+^ T cell agonism, resulting in immune activation by expansion and boosting of effector cells.

So, what is the common denominator determining FcγR-dependency, and preferential engagement of inhibitory vs. activating FcγR for efficacy of individual targets and antibodies? In a recent landmark paper, Beers and co-workers used a multi-pronged approach to study molecular and cellular FcγR-dependent mechanisms underlying therapeutic activity of antibodies to the co-stimulatory immune checkpoint 4-1BB (89). Firstly, the authors used anti-4-1BB antibodies with identical Fv-regions but differing in isotype–therefore targeting the same epitope on 4-1BB but showing preferential engagement of activating (mouse IgG2a, high A:I ratio) or inhibitory (mouse IgG1, low A:I ratio) antibody checkpoints. Second, effects were studied in immunocompetent tumor-bearing animals differing only by FcγR repertoire–expressing only activating, only inhibitory or both activating and inhibitory antibody checkpoints. Using this approach, the authors found that anti-4-1BB antibodies can stimulate anti-tumor immunity by different mechanisms; Boosting of effector CD8^+^ T cells, or depletion of regulatory T cells (Figure 4). Both mechanisms were regulated by antibody interactions with FcγR, but differently so.

Anti-4-1BB antibodies' depletion of intratumoral Treg cells was shown to be dependent on activating FcγR (89). Antibody isotypes with high A:I ratio showed enhanced Treg deletion, and Treg deletion was diminished in animals lacking activating Fc gamma receptors. A similar dependence on activating antibody checkpoints for Treg depletion had previously been demonstrated for antibodies to other immune receptors e.g., GITR, OX40, CD40, CTLA-4, or IL-2R, i.e., independent of specificity for co-stimulatory or inhibitory immune checkpoints (Table 1).

Conversely, boosting of CD8^+^ T cell responses was most pronounced with antibody isotypes of low A:I ratio. The mechanism underlying enhanced CD8^+^ T cell responses likely involves FcγRIIB-mediated antibody cross-linking, and thereby promoted signaling, of antibody-targeted co-stimulatory 4-1BB receptors on CD8^+^ T cells. Agonist anti-tumor activity of anti-CD40 antibodies has previously been proposed to rely on FcγRIIB-mediated antibody cross-linking and promoted signaling in CD40-expressing antigen presenting cells (23, 24) (Table 1; Figure 2B).

Interestingly, the authors found that concurrent administration of equal doses of high A:I variant (mIgG2a), Treg-depleting, anti-4-1-BB antibodies, and low A:I variant (mIgG1), CD8^+^ T cell boosting, anti-4-1-BB antibodies reduced therapeutic efficacy. In contrast, sequential administration of first activating FcγR-optimized antibody to deplete Tregs, followed by inhibitory FcγR-optimized antibody to agonize CD8^+^ T cells, enhanced therapeutic efficacy compared to single agent treatment. These observations indicated competing mechanisms of high A:I antibody mediated Treg depletion, and low A:I antibody mediated CD8^+^ T cell boosting. This notion that was corroborated through a series of complementary experiments. In short, although the two studied isotype variant antibodies show preferential binding to activatory (mIgG2a, high A:I ratio) and inhibitory (mIgG1, low A:I ratio) FcγRs, respectively, both antibody variants will co-engage activating and inhibitory FcγRs *in vivo*, where their “preferred” type (activating or inhibitory) of FcγR on effector cells is limited in numbers, relative to target cell coated antibody Fc's available for FcγR engagement. Therefore, concurrently administered high A:I ratio and low A:I ratio antibodies will compete for binding to available activating and inhibitory FcγR, resulting in a “frustrated system” of suboptimal Treg depletion and suboptimal CD8^+^ T cell boosting.

Importantly, if translated to human, these findings could have broad implications for cancer immunotherapy. Human IgG1 and IgG4 antibodies–two of the most common isotypes used in cancer immunotherapy–bind human activating and inhibitory FcγRs with rather similar affinity, compared with the more “polar” affinities of mIgG2a and mIgG1 for activating, and inhibitory FcγRs, respectively. Human IgG1 and IgG4 might therefore be expected to be quite sensitive to such competition, which could help explain the poor translation of promising mouse data to the human clinical setting. Further, the findings are likely relevant to other signaling antibody targets, most notably co-stimulatory receptors of the TNF receptor superfamily. Earlier studies had reported decreased efficacy following concurrent treatment with antibodies to OX40 and PD-1, although underlying molecular mechanisms were not studied (88).

**Figure 5:**
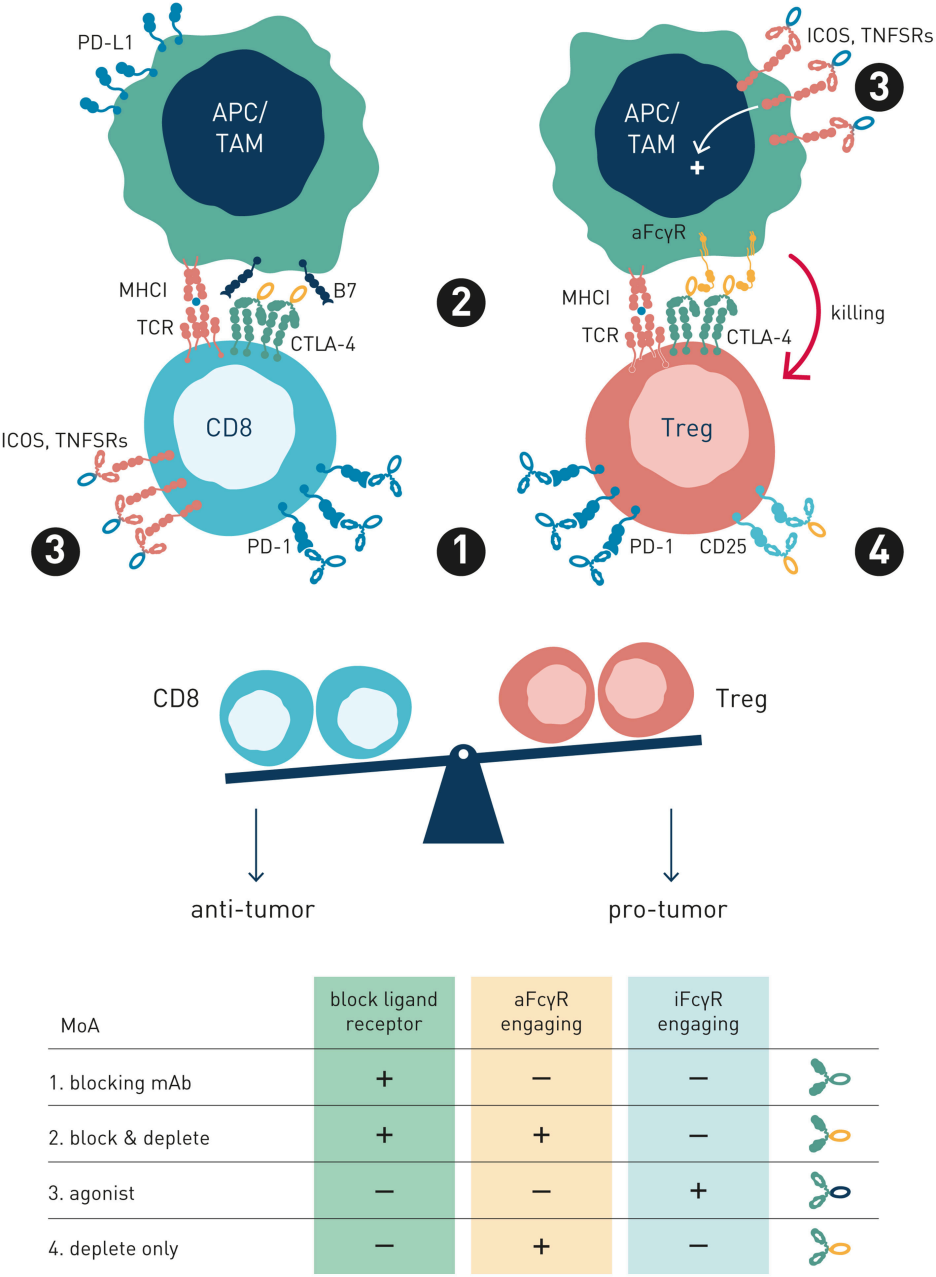
Antibody preferred mechanism-of-action and FcγR-engagement is dependent on target receptor function and expression. This schematic figure models four exemplary antibody MoA's, pertinent to both immune checkpoint and tumor cell direct-targeting antibody types. (1) Blocking mAb. PD-1, a co-inhibitory antibody checkpoint expressed at high and similar levels on intratumoral Treg and Teff cells, is best targeted using a PD-1/PD-L1 blocking Fc-null antibody variant, since FcγR-mediated Teff cell depletion is undesirable (2) Blocking and depleting mAbs. Anti-CTLA-4 is overexpressed on intratumoral Treg compared with Teff, and activatory FcγR-engagement correlates with survival in melanoma patients treated with ipilimumab. Preferred MoA is two-fold: CTLA-4/B7 blockade and Treg depletion through FcγR-dependent mechanisms (3) Agonist mAb Preferred MoA is FcγR-engaging antibody variant, where FcγRs promote receptor cross-linking and signaling. (4) Depletion only mAb. Anti-IL2R antibody preferred MoA is ligand non-blocking and FcγR-dependent (Treg) cell depletion. IL-2R overexpressing Tregs are selectively depleted, while free IL-2 may promote Teff survival and expansion.

Collectively, these observations shed important light on how antibody checkpoints regulate mechanisms common to cancer cell direct-targeting and immune checkpoint targeting antibodies. Therapeutic activity of either type of antibody may rely principally on target cell depletion (e.g., anti-CD20 or anti-IL-2R), cell depletion and block of target receptor signaling (e.g., anti-Her2 or anti-CTLA-4), or strictly on receptor/ligand blockade e.g., anti-PD-1 (Figure 5). Thus, classification of antibodies into cancer cell-direct targeting, immune checkpoint blocking, or immune checkpoint agonists, is inadequate and needs revision (98). Instead, careful dissection of individual antibodies' mechanism(s) of action with respect to their ability to block or agonize receptor signaling and/or deplete target cell(s), and their regulation by interactions with the antibody checkpoints, will be critical for identification and rational combination of antibodies with complementary non-competing mechanisms-of-action (Table 1). As discussed below, such knowledge will additionally pave the way for antibody-checkpoint targeted therapies, e.g., antibody blockade of inhibitory FcγRIIB or Fc-engineering for enhanced affinity to activating FcγR, to help boost efficacy and overcome resistance in the immune suppressed tumor microenvironment.

### Targeting the Antibody Checkpoints to Improve Cancer Immunotherapy–Focus on FcγRIIB

The documented role of the antibody checkpoints as master regulators of the clinically most relevant classes of anti-cancer antibodies detailed above, suggests that targeting of this receptor family be an attractive strategy to enhance efficacy and overcome resistance to antibody-based cancer immunotherapy.

While Fc gamma receptor regulation of antibody efficacy is highly functionally conserved between mouse and man, important differences in absolute and relative binding affinities of the species' respective antibody subclasses for their corresponding activating and inhibitory FcγRs have slowed translation into human therapeutic antibody candidates and clinical development. Recent development of FcR-humanized mouse models (99), and highly specific antagonist or agonist antibodies to individual human and mouse activating and inhibitory receptors (30, 65), have now enabled such translation.

**Figure 6:**
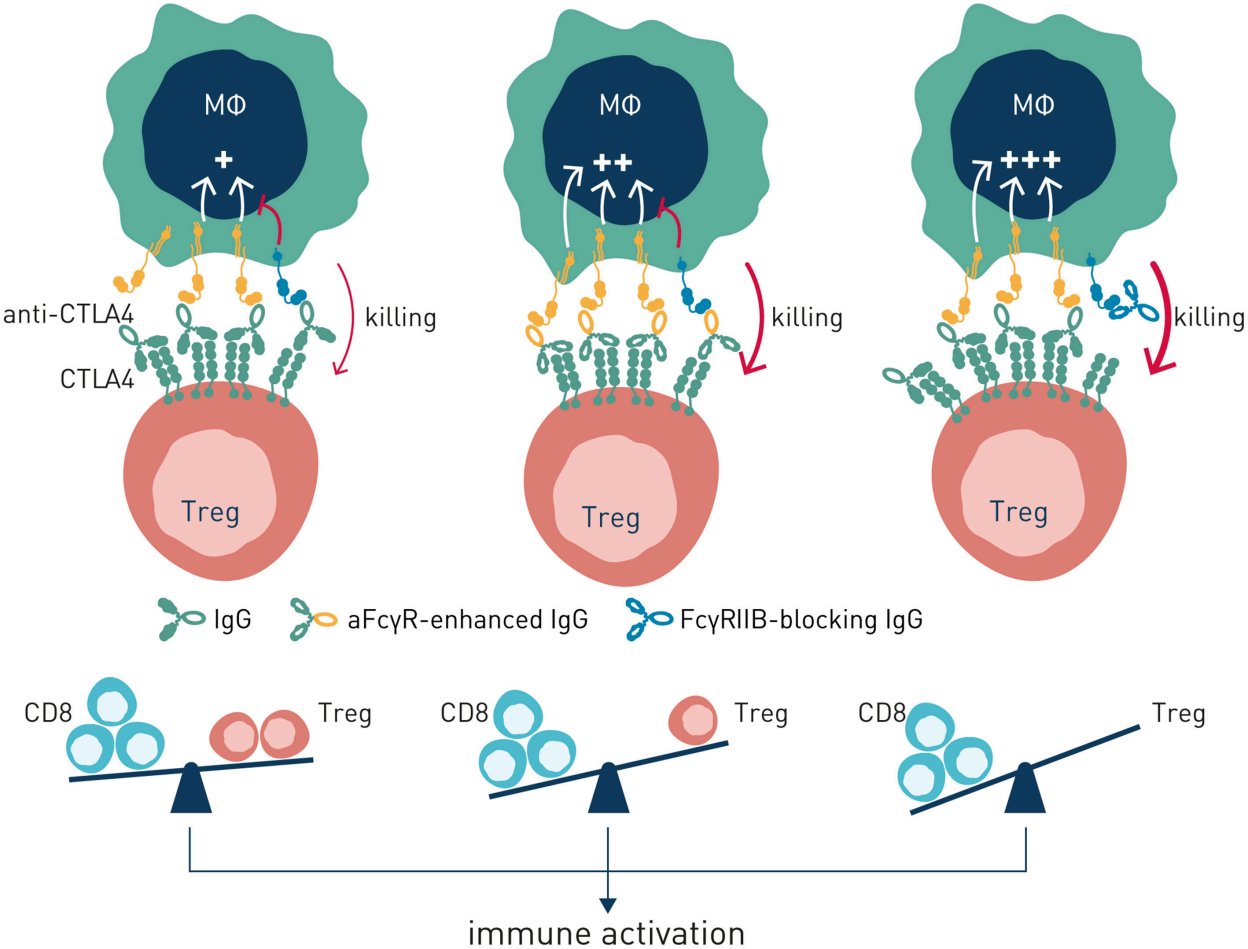
Antibody-induced antitumor immunity can be enhanced by modulation of antibody: FcγR interactions. **Left** panel (no antibody checkpoint modulation). Antibody efficacy is balanced by co-engagement of activating and inhibitory FcγR. **Center** panel (enhanced engagement of activating FcγR). Antibody efficacy is improved through Fc-engineering for enhanced binding to activating FcγR. **Right** panel–Antibody efficacy is enhanced by blockade of the inhibitory FcγRIIB.

Two principal strategies to better harness antibody checkpoint-dependent antitumor immunity have been pursued–Fc engineering or FcγR blockade (Figure 6). Antibody engineering to enhance affinity for activating antibody checkpoints has obtained clinical proof-of-concept through the afucosylated CD20-specific antibody obinutuzumab (15), with additional afucosylated antibodies in late stage clinical development (100). While clinically validated, and elegant in the sense that simple removal of a fucose group of residue N297 in the antibody constant domain results in very significantly enhanced binding to FcγRIIIa (101), this approach has its limitations. Firstly, emerging data indicates that intratumoral macrophages and dendritic cells–critical effectors underlying antibody-induced antitumor immunity (102)–express FcγRIIA and FcγRIIB at highest density (76). Further, FcγRIIA may be the only activating Fc gamma receptor expressed on human dendritic cells, which additionally express FcγRIIB for coordinate regulation of antigen presentation (45). Consequently, harnessing the full potential of antibody checkpoint-regulated anti-cancer immunity is likely to require engagement and enhancement of additional activating FcγRs besides FcγRIIIa, and ideally reduced or no engagement of the inhibitory antibody checkpoint. As discussed below, the great structural similarity between individual activating and inhibitory antibody checkpoint receptors poses significant technical challenges to succeed in engineering of antibodies with such properties. Nevertheless, Fc-engineering by substitution of two or more amino acids has generated antibody molecules with enhanced affinity for both FcγRIIA and FcγRIIIA, albeit with retained or slightly enhanced affinity also for the inhibitory FcγRIIB (103, 104). Whether such molecules will show therapeutically relevant pharmacokinetics or enhanced efficacy remains to be demonstrated in clinical trials.

Based on the significant upregulation of the sole inhibitory antibody checkpoint FcγRIIB in the tumor microenvironment (97), and its documented role in conferring resistance to antibody-based therapy in this niche (65, 70, 97), we have pursued antibody-mediated blockade of FcγRIIB as an alternative and complementary approach to Fc-engineering to harness the full potential of antibody checkpoint-regulated immunity. In theory, besides being an apparent critical pan-antibody regulator conferring antibody drug resistance in the tumor microenvironment, targeted blockade of FcγRIIB by a separate antibody has the advantage of enabling combination therapy and boosted efficacy with multiple existing, clinically validated, antibodies including those engineered for enhanced binding to activating FcγR (65). The strategy does, however, put exquisite requirements on a therapeutic antibody candidate, both from target receptor specificity and function-modulating perspectives. The extracellular, antibody accessible domain, of the inhibitory FcγRIIB is ~93% homologous with the activating FcγRIIA. Nevertheless, probing of a highly diversified human recombinant antibody library (65), or immunization of mice transgenic for human FcγRIIA (105), generated diverse pools of highly specific antibodies that selectively bound to FcγRIIB, and not to FcγRIIA, and which in a dose-dependent manner blocked immune complex binding to cell surface-expressed FcγRIIB. Functional screening revealed that only a minority of the highly FcγRIIB specific human recombinant antibodies were able to block antibody-induced FcγRIIB inhibitory signaling (65). Remaining candidates either did not block, or agonized, FcγRIIB signaling. The latter category could have therapeutic potential in treatment of chronic inflammatory and autoimmune disease (106).

Based on observations that FcγRIIB limits antibody efficacy and promotes tumor cell resistance by dual mechanisms in B cell malignancy, acting at the level of both immune effector cells and tumor B cells, we have further characterized the therapeutic potential of antagonistic anti-FcγRIIB antibodies to boost efficacy and overcome resistance to antibody therapy *in vivo* focusing initially on this setting. A lead human antagonistic anti-FcγRIIB IgG1 antibody (6G11 or BI-1206), which showed synergistically enhanced rituximab B cell depletion in FcγRIIB and CD20 humanized mice, and overcame refractoriness of primary leukaemic B cells to anti-CD20-based antibody therapy *in vivo*, is currently in early phase clinical testing (65).

Besides affording efficacy, therapeutic targeting of Fc gamma receptors, whether by blocking antibodies or Fc-engineering, must be safe and associated with therapeutically relevant pharmacokinetics. In addition to its high expression on B cells and certain macrophage/dendritic cells, FcγRIIB has been reported to be highly expressed in mouse and rat liver sinusoidal endothelial cells (LSEC) (107), where they have been implicated in removal of circulating small immune complexes (108). These observations raise potential safety concerns of undesirably cytotoxic activity with therapeutic antibodies targeting FcγRIIB. However, our recent observations of human and mouse liver indicate lower LSEC expression in man (30), and dosing of FcγRIIB humanized mice with therapeutically relevant doses of anti-human FcγRIIB IgG1 antibody 6G11 showed no apparent acute or chronic treatment related adverse effects (30, 65). Ultimately, the safety and efficacy of targeting FcγRIIB needs to be assessed in human subjects. Two clinical trials are ongoing to evaluate safety and explore efficacy of the BI-1206 antibody as single agent and in combination with rituximab in B cell malignancy (NCT03571568 and NCT02933320). Our ongoing efforts aim at translating observations of FcγRIIB-regulated antitumor immunity to the solid cancer clinical setting.

As noted above FcγRIIB may promote anti-tumor activity by facilitating extrinsic signaling of certain co-stimulatory receptors expressed on tumor or immune cells. A possible strategy to enhance therapeutic activity of such antibodies would therefore be to enhance their affinity for FcγRIIB. In keeping with this, anti-DR5 antibodies carrying the S267E (“SE”) mutation, increasing human IgG1 affinity for FcγRIIB several hundred-fold, showed improved tumor regression in mouse models humanized for FcγRIIB (109). Analogously, human IgG2 anti-CD40 antibodies equipped with SE or SE/LF mutated backbones (the latter further increases affinity for FcγRIIB) showed enhanced CD8^+^ T cell activation, and improved ability to clear tumors, in mice humanized for FcγRs and CD40 (96). However, increasing antibody affinity for FcγRIIB in these two cases improved not only efficacy but also side effects. Increased DR5 agonism of the SE variant anti-DR5 was associated with increased liver enzyme release. SE and SE/LF variant anti-CD40 antibodies increased not only T cell activation and anti-tumor immunity, but also depletion of platelets, which express CD40 (96, 109). Thus, Fc-engineering for enhanced FcγRIIB affinity or selectivity needs close consideration of antibody (Fv-) targeted receptor's cellular distribution and function(s).

## Concluding Remarks

Emerging preclinical and clinical data demonstrate that the activating and inhibitory Fc gamma receptors–the “antibody checkpoints”–control antitumor immunity induced by the clinically most successful antibodies used in cancer immunotherapy. Therapeutics that harness the power of antibody checkpoint-regulated anti-tumor immunity, through Fc-engineering to enhance binding to activating FcγRs, or through blockade of the inhibitory FcγRIIB, have been approved or are in development. If safe and well-tolerated, these agents hold promise to improve response rates, duration of response, and potentially overall survival for diverse cancer patients.

## Author Contributions

BF wrote and edited the manuscript and conceived figures. IT helped write the manuscript and conceive figures. LM designed and performed experiments in several of herein reviewed papers, and helped write the current manuscript and helped conceive figures.

### Conflict of Interest Statement

IT, LM, and BF are employees of and hold stock in BioInvent (www.BioInvent.com), a company developing antibody-based cancer immunotherapeutics, including anti-FcγRIIB antibodies.
